# Non-invasive biomarkers for spontaneous intracranial hypotension (SIH) through phase-contrast MRI

**DOI:** 10.1007/s00415-024-12365-6

**Published:** 2024-04-21

**Authors:** Katharina Wolf, Florian Volz, Niklas Lützen, Hansjoerg Mast, Marco Reisert, Amir El Rahal, Christian Fung, Mukesch J. Shah, Jürgen Beck, Horst Urbach

**Affiliations:** 1 Department of Neurosurgery, Medical Center, Faculty of Medicine, University of Freiburg, Breisacher Str. 64, 79106 Freiburg, Germany; 2 Department of Neuroradiology, Medical Center, Faculty of Medicine, University of Freiburg, Freiburg, Germany; 3 Department of Radiology, Medical Physics, Medical Center, Faculty of Medicine, University of Freiburg, Freiburg, Germany; 4 Department of Stereotactic and Functional Neurosurgery, Medical Center, Faculty of Medicine, University of Freiburg, Freiburg, Germany; 5 Department of Neurosurgery, Faculty of Medicine, University of Geneva, Geneva, Switzerland

**Keywords:** Spontaneous intracranial hypotension, Phase-contrast MRI, Spinal cord motion, CSF flow, CSF–venous fistula

## Abstract

**Background and objective:**

Spontaneous intracranial hypotension (SIH) is an underdiagnosed disease. To depict the accurate diagnosis can be demanding; especially the detection of CSF–venous fistulas poses many challenges. Potential dynamic biomarkers have been identified through non-invasive phase-contrast MRI in a limited subset of SIH patients with evidence of spinal longitudinal extradural collection. This study aimed to explore these biomarkers related to spinal cord motion and CSF velocities in a broader SIH cohort.

**Methods:**

A retrospective, monocentric pooled-data analysis was conducted of patients suspected to suffer from SIH who underwent phase-contrast MRI for spinal cord and CSF velocity measurements at segment C2/C3 referred to a tertiary center between February 2022 and June 2023. Velocity ranges (mm/s), total displacement (mm), and further derivatives were assessed and compared to data from the database of 70 healthy controls.

**Results:**

In 117 patients, a leak was located (54% ventral leak, 20% lateral leak, 20% CSF–venous fistulas, 6% sacral leaks). SIH patients showed larger spinal cord and CSF velocities than healthy controls: e.g., velocity range 7.6 ± 3 mm/s vs. 5.6 ± 1.4 mm/s, 56 ± 21 mm/s vs. 42 ± 10 mm/s, *p* < 0.001, respectively. Patients with lateral leaks and CSF–venous fistulas exhibited an exceptionally heightened level of spinal cord motion (e.g., velocity range 8.4 ± 3.3 mm/s; 8.2 ± 3.1 mm/s vs. 5.6 ± 1.4 mm/s, *p* < 0.001, respectively).

**Conclusion:**

Phase-contrast MRI might become a valuable tool for SIH diagnosis, especially in patients with CSF–venous fistulas without evidence of spinal extradural fluid collection.

**Supplementary Information:**

The online version contains supplementary material available at 10.1007/s00415-024-12365-6.

## Introduction

Spontaneous intracranial hypotension (SIH) is an underdiagnosed disease caused by spinal CSF leaks, manifesting at any age [1]. The annual incidence rate of 4–5/100,000 is estimated [2–4]. Typically, clinical manifestation entails, but is not limited to the onset of a new orthostatic headache [1, 5, 6]. Other symptoms comprise audiovestibular deficits, visual disturbances, nausea, and cognitive decline, among an array of many other complaints [7]. The clinical presentation can appear inconsistent or even paradoxical, posing diagnostic challenges [8], and the patient at risk of chronic subdural hematoma [9–12], bibrachial amyotrophy [13], mimics of fronto-temporal dementia [14–16], and deficits caused by superficial siderosis [13, 17–19].

SIH might be caused by different types of leaks that are commonly classified into three types: ventral (type 1), lateral (type 2), and CSF–venous fistulas (type 3) [20, 21]. Additionally, a leak might be found at the sacrum (sacral leaks) [22]. MRI findings serve as an important diagnostic tool for SIH screening. The so-called Bern score [23] evaluates and rates several signs in the contrast-enhanced MRI of the head indicative of spinal CSF loss. Nevertheless, a normal Bern score does not conclusively rule out a spinal CSF leak. The presence of spinal longitudinal extradural collections (SLEC) proves a spinal CSF leak, and additionally signifies ventral (type 1) or lateral CSF (type 2) leaks that drain into the epidural space. However, a notable proportion of SIH patients with CSF–venous fistulas (type 3), which account for about 20–25% of patients and increasing, present without SLEC. Hence, determining the next steps for such patients negative for SLEC and without clear signs in the MRI of the brain remains a challenge.

Non-invasive, cardiac-gated phase-contrast MRI at the upper cervical spine has been introduced as a novel approach to gain comprehensive, dynamic data on spinal cord, and CSF velocities in SIH [24]. An increase in the overall dynamics at the upper cervical spine has been demonstrated in SIH patients positive for SLEC that has been hypothesized to reflect a reduced resistance due to CSF loss within the craniospinal compartments. This study aimed to reassess these previous findings and the possible diagnostic value in a larger cohort, encompassing various spinal CSF leaks associated with SIH.

## Methods

This study reports a monocentric, retrospective, controlled, pooled-data analysis involving patients admitted to a tertiary referral center between February 2022 and June 2023 with suspected or previously confirmed SIH [7], and who underwent full SIH MRI workup according to center-specific standards including dynamic, ECG-gated phase-contrast MRI as of February 2022. Suspected cases of SIH were identified based on patient history indicative of SIH, mainly characterized by newly onset orthostatic headaches and/or signs of spinal CSF leaks in an earlier MRI of the head or spine (according to the Bern score ± SLEC). Additionally, patient data was pooled with previously published, prospective data of 20 SIH patients positive for SLEC assessed between October 2021 and February 2022 [24]. The entire pooled data was then compared to data of 70 healthy controls from the database (age 20–79 years, mean age 45.2 ± 16 years, 56% women) [25]. The local ethical board granted approval for the prospective trial (vote number: 338/17), and the subsequent retrospective analysis (vote number: 22-1512_1-S1-retro). Patients and healthy participants from the prospective trial provided individual consent prior to participation. As per ethical board decision, an individual consent was not required for the retrospective investigation.

### MRI

**Fig. 1:**
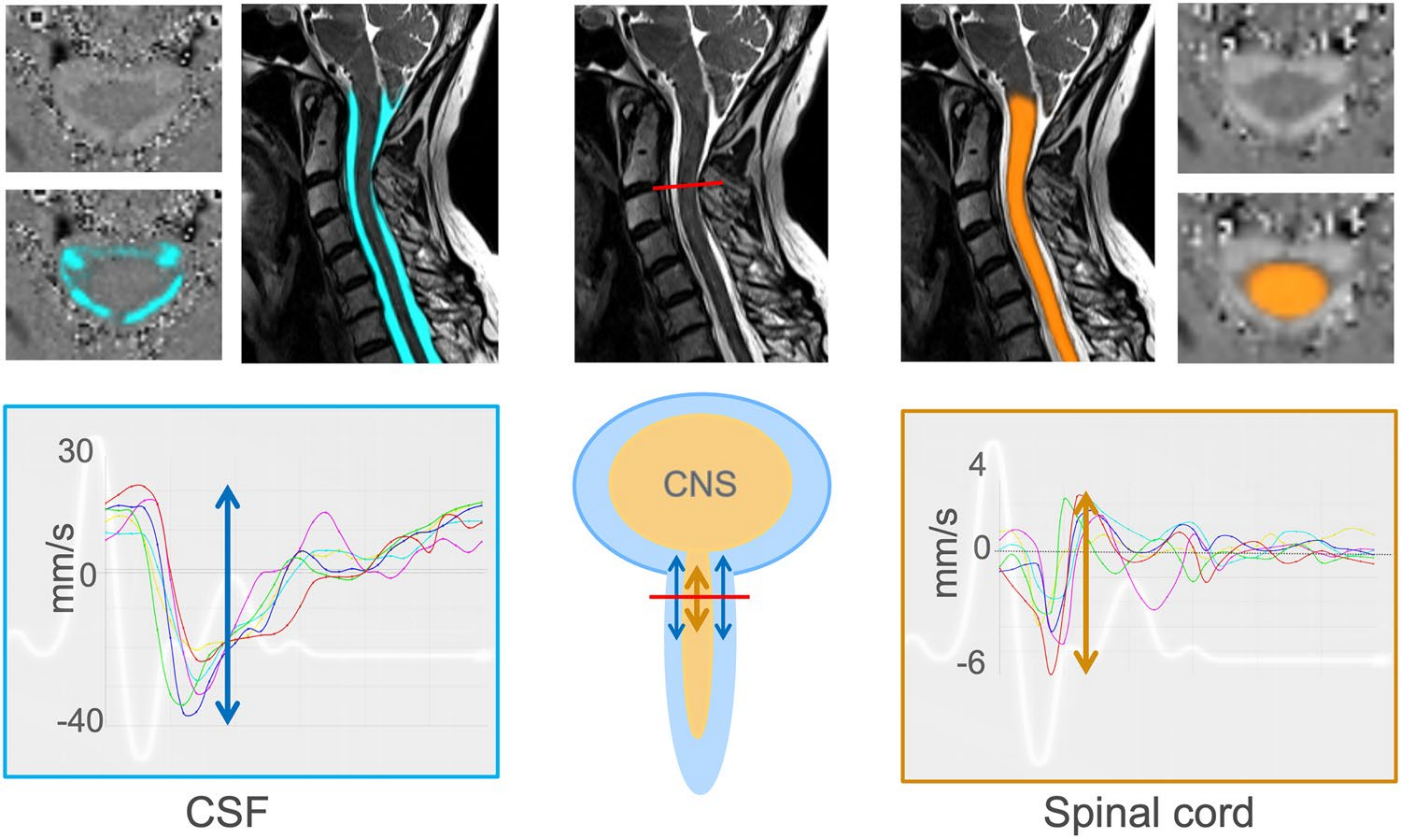
Examples of MRI sequences and MRI processing among patients with SIH—top row with sagittal 3D T2 SPACE of the cervical spine, without (middle) and with segmentation (blue = CSF, left, orange = spinal cord, right side). The axial images are phase images with and without automated segmentation. Through-plane axial measurements at C2/C3 (red line) encode velocities in head–feet direction (arrows). At the bottom, acquired velocities in mm/s (y-axis) are plotted over one standardized heartbeat (x-axis). Measurements start with the R-peak of the ECG. Each colored line indicates a different individual. The arrows within the time-resolved plots indicate the peak-to-peak velocity amplitude = velocity range in the craniocaudal direction

All participants were scanned on a 3 Tesla MRI scanner (3 T, SIEMENS MAGNETOM Prisma, SIEMENS Healthineers, Erlangen) using a 64-channel head–neck coil. Phase-contrast MRI was performed as part of the standard diagnostic workup in SIH that also includes contrast-enhanced MRI of the head to assess the Bern score, and T2 fat-saturated images of the entire spine to screen for SLEC, prior to dynamic myelograms [26]. Standard T2 3D sequence of the cervical spine (spatial resolution 0.6 mm × 0.6 mm × 1.0 mm) was used to derive anatomical data of the CSF space and the spinal cord cross-sectional areas (CSA) (Fig. 1). The phase-contrast imaging protocol comprised two axial, cardiac-gated 2D phase-contrast MRI sequences that were administered during free, steady breathing for through-plane cranio-caudal velocities (prospective ECG-triggering, spatial resolution 0.9 mm × 0.9 mm x 5 mm). The sequences were set perpendicular to the spinal canal at the cervical segment C2/C3 with velocity-encoding parameter 5 cm/s for spinal cord velocities and velocity-encoding parameter 10 cm/s for CSF velocities. A minimum of 20 time points per cardiac cycle was assessed per sequence. The average time of the individual’s heartbeat duration was recorded per sequence allowing further analysis of the time-resolved velocity data (about 1.5 min). Further technical MRI details are summarized in Supplement 1.

Data segmentation and processing were performed using a fully automated pipeline implied within the in-house platform NORA (ww.nora-imaging.org [27]) that uses trained 3D hierarchical deep convolutional neural networks for segmentation (Fig. 1). The training procedures and the further processing steps have been described in detail before [24, 25], and are summarized in Supplement 1. MRI data curation involved exclusion of datasets with typical MRI artifacts (e.g., movement, metal, infolding), and/or exclusion of velocity curves that did not follow a typical pattern of CSF- or spinal cord motion as displayed in Fig. 1 (examples in Supplement 1). Data validity of the methods regarding test–test and test–retest reliability has been investigated and reported before (Supplement 1) [24, 25]. The Intra-class correlation coefficient was > 0.9 for any value.

### Parameter of interest

The following parameter were assessed: sex, age (years), height (cm), body mass index (BMI) (kg/m^2^), duration of symptoms (months), and prior treatment with epidural lumbar bloodpatch (EBP), targeted fibrin patch, and surgery. Among patients, the Bern score [23] and presence of SLEC were recorded. Bern score was additionally stratified into low (≤ 2 points), intermediate (3–4 points), and high probability (5–9 points) [23]. The type of the CSF leak [20–22] was diagnosed on subsequent dynamic myelogram (digital subtraction myelogram and/or dynamic CT-myelogram).

For anatomical data of the cervical spine, the spinal cord cross-sectional area (CSA) (mm^2^) and the subarachnoid space CSA (mm^2^) were generated per segment using the T2-weighted images.

For quantitative analysis of the time-resolved velocity curves, two main values were calculated (Fig. 1): the maximum craniocaudal peak-to-peak amplitude that comprises the velocity range (mm/s), and the total displacement (mm) that refers to the absolute distance that the chosen voxels are moved up and down per heart cycle. In addition, the maximum range of the CSF flow rate in ml/s and the CSF stroke volume (ml) was generated. As significant influences of age (negative effect), sex (increased values in males), and for CSF space narrowing of the spinal canal at the level C2/C3 (positive effect for CSF only) have been evident in healthy cohorts [25], dynamic data was additionally adjusted accordingly and reported in arbitrary units.

### Statistics

Statistical analysis was performed using IBM SPSS Statistics® (IBM Corporation, Released 2020. IBM SPSS Statistics for Macintosh, Version 27.0. Armonk, New York, USA).

Data are presented as mean and standard deviation (SD), unless stated otherwise.

Data of (1) all patients with SIH and confirmed spinal CSF leak, (2) subgroups divided by spinal CSF leak type, and (3) of all SIH patients with confirmed spinal CSF leak *and* Bern score of 0–4 points were compared to healthy controls by Mann–Whitney *U* test. Distribution of categorical data was compared via *χ*^2^ fisher exact test. Furthermore, subgroups stratified by spinal CSF leak types were compared using the Kruskal–Wallis test, requiring a minimum of ten cases per leak type.

Among all SIH patients with confirmed spinal CSF leak, the relation of duration of symptoms and of the Bern score (0–9 points) with adjusted phase-contrast MRI measurements were analyzed by multiple linear regression upon validation of standard premises applying bootstrapping to adjust for optimism. As the duration of symptoms showed a right skew, data were converted to a logarithmic scale beforehand. P < 0.05 was considered significant.

Receiver operating characteristics (AUROC) analysis was conducted for all SIH patients with confirmed spinal CSF leaks to assess the diagnostic potential of phase-contrast MRI analysis in SIH. Thresholds were selected to achieve ≥ 90% specificity. Additionally, sensitivity and specificity of these thresholds were separately analyzed in all patients with CSF–venous fistulas.

Descriptive reporting was provided for patients’ results in these cases where the diagnosis remained unclear or no spinal CSF leak was found at the time of the analysis.

## Results

**Fig. 2:**
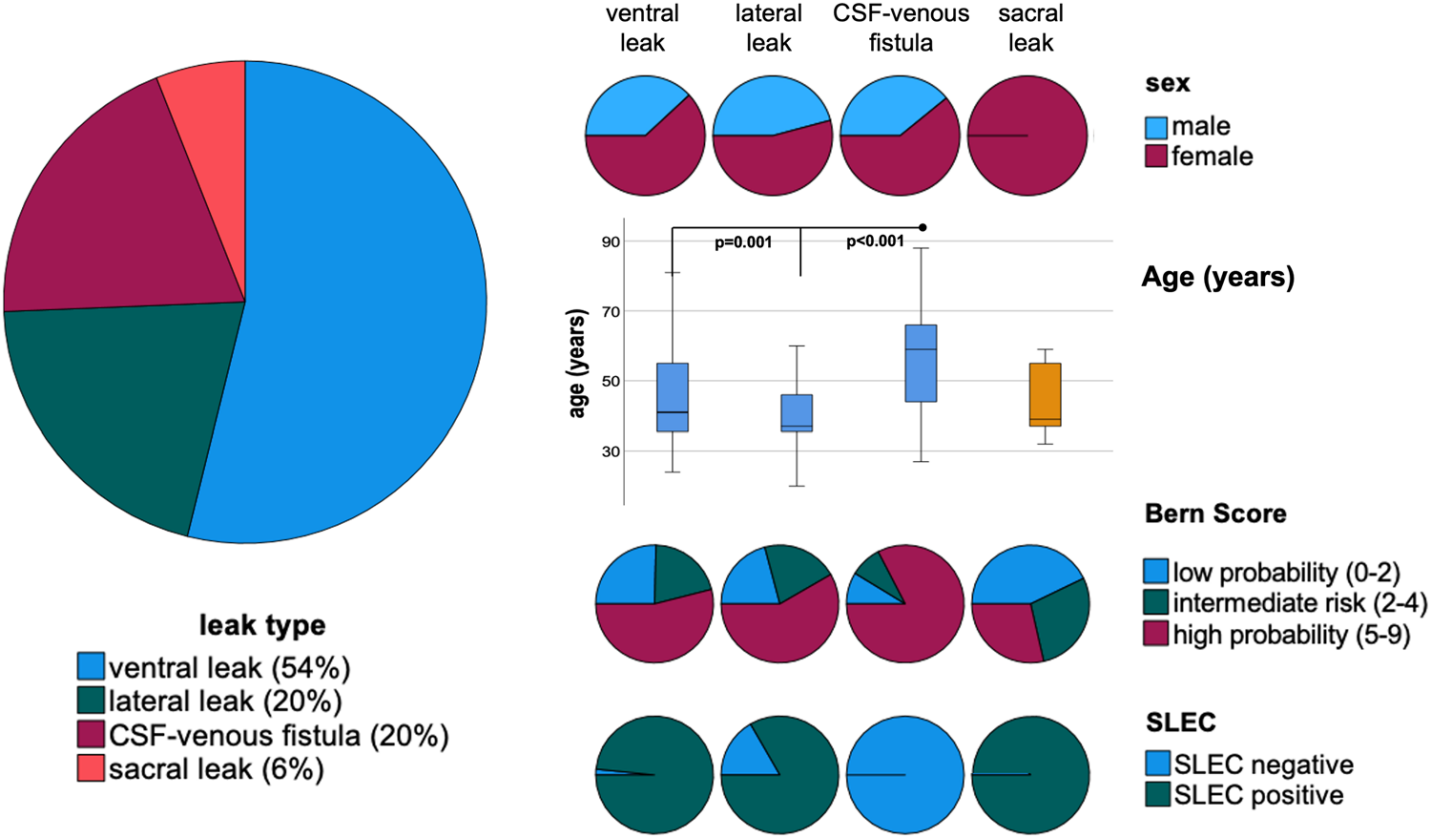
Clinical data of patients with confirmed spinal CSF leaks, *n* = 117, spinal longitudinal extradural collection (SLEC). Patients with CSF–venous fistulas were older than patients with ventral or lateral leaks (*p* < 0.001, *p* = 0.001, respectively) and showed higher Bern scores (*p* > 0.082)

A total of 125 patients were identified, with 59% being female (Table 1, Fig. 2). Among these individuals, 117 had a confirmed spinal CSF leak through myelography and were included in the main analysis.

**Table 1:** study population, epidural blood patch (EBP), spinal longitudinal extradural collection (SLEC)

Total, *n* = 125	Confirmed spinal CSF leak	Ventral leak	Lateral leak	CSF–venous fistula	Sacral leak	No leak confirmed
Number	117	63	24	23	7	8
Male/female (%)	54/73 (38/62%)	24/39 (39/61%)	11/13 (46/54%)	9/14 (40/60%)	0/7 (0/100%)	4/4 (50/50%)
Age (years) (range)	46 ± 14 (20–88)	44 ± 13 (24–81)	40 ± 10 (20–60)	57 ± 16* (27–88)	45 ± 11 (32–59)	54 ± 15 (30–80)
Bern score (range)	5.1 ± 3 (0–9)	4.8 ± 3 (0–9)	5.4 ± 3 (1–9)	6.0 ± 2 (1–9)	3.7 ± 2 (2–7)	3.3 ± 2 (0–5)
Low risk (≤ 2 points)	26 (22%)	16 (25%)	5 (21%)	2 (9%)	3 (42%)	2 (25%)
Intermediate risk (3–4 points)	22 (19%)	13 (21%)	5 (21%)	2 (9%)	2 (29%)	4 (40%)
High risk (5–9 points)	69 (59%)	34 (54%)	14 (58%)	19 (82%)	2 (29%)	2 (25%)
SLEC positive	90 (72%)	62 (98%)	20 (83%)	0	7 (100%)	0**
Duration of symptoms in months (range)	15 ± 30 (0.2–179)	13 ± 31 (0.2–180)	21 ± 36 (0.2–132)	19 ± 24 (0.3–84)	6 ± 5 (0.5–13)	9 ± 12 (0.5–36)
Prior invasive therapy (%)	57 (49%)	28 (44%)	15 (63%)	12 (52%)	2 (29%)	3 (38%)
Lumbar EBP (%)	52 (44%)	27 (43%)	13 (54%)	10 (43%)	2 (29%)	3 (38%)
Targeted fibrin patch (%)	1 (0.8%)	/	1 (4%)	0	0	/
Embolization (%)	2 (1.6%)	/	/	2 (9%)	/	/
Surgery (%)	2 (1.6%)	1 (2%)	1 (4%)	/	0	0
Excluded measurements: spinal cord/CSF	10/22	7/13	1/3	2/4	0/2	0/0

*Patients with CSF venous fistulas were significantly older than patients with ventral and lateral leaks (*p* = 0.001, *p* < 0.001, respectively)

**One patient showed evidence of SLEC in the MRI of the spine before admission

The breakdown of spinal CSF leak types included 63/117 (54%) with ventral leaks (Type I), 24/117 (20%) with lateral leaks (Type II), 23/117 (20%) with CSF–venous fistulas (Type III), and 7/117 (6%) with sacral leaks (Fig. 2). Within this group, 27 were rated negative for SLEC before myelography (23%, one patient with ventral leak, three with lateral leaks, all 23 patients with CSF–venous fistulas). Post-confirmation of the leak site, a small SLEC was retrospectively identified in the patient with ventral leak, located at the site of a calcified disc protrusion. Two patients with lateral leaks negative for SLEC had received targeted fibrin patch or surgical treatment in advance.

Of the eight remaining patients, one exhibited spontaneous resolution of SLEC and elevated Bern score in the MRI beforehand, while in seven patients, the diagnosis of SIH was not confirmed (Table 1).

When categorized by leak type, the duration of symptoms was similar. Notably, all patients with sacral leaks were female. Otherwise, the distribution of sex ranged similarly, with a female predominance (54–61%). Comparison of the Bern score between leak types did not yield statistical significance (Kruskal–Wallis test *p* > 0.082, *χ*^2^ *p* > 0.053), although a tendency toward a higher Bern score was observed among patients with CSF–venous fistulas. Additionally, patients with CSF–venous fistulas (57 ± 16 years) were older compared to patients with ventral (44 ± 13 years, *p* = 0.001) and lateral leak (40 ± 10 years, *p* < 0.001). Segmental narrowing of the spinal cord (aSCOR) was not different between leak types. No patient showed severe spinal canal narrowing > 60%, while three patients with ventral leak showed > 50% narrowing at the segment C5/C6 (supplement 2). 49% of all patients had received prior therapy for invasive SIH (Table 1).

Healthy participants (56% female) showed a mean age of 45 ± 16 years (range 21 to 79 years). Sex distribution and age did not differ between healthy participants and patients with confirmed leak (*p* = 0.441, *p* = 0.537). Also, the height and body mass index were not different (patients 1.73 ± 0.1 m, 24.2 24.2 ± 4 kg/m^2^, healthy participants 1.73 ± 0.1 m, 24.1 ± 3 24.2 ± 4 kg/m^2^, *p* = 0.221, *p* = 0.831, respectively). Segmental narrowing at the craniospinal junction C2/C3 and C3/C4 was more pronounced among patients (aSCOR C2/C3 28.3 ± 5.7% vs 24.0 ± 4.1%, *p* < 0.001, aSCOR C3/C4 32.8 ± 5.3% vs. 29.9 ± 5.0%, 0.011, complete data in supplement 2).

About 10% of the spinal cord and about 20% of the CSF measurements were excluded prior to statistic analysis (ventral leak: 7 / 13, lateral leak 1 /3, CSF–venous fistula 2 /4, sacral leak 0/2, patients without confirmed leak 0 / 0, healthy controls 5/6, respectively).

### Cardiac-related spinal cord and CSF velocities

SIH patients with confirmed leaks exhibited elevated spinal cord and CSF velocity ranges, increased total spinal cord and CSF displacements, and increased ranges of CSF flow rates compared to healthy controls (Table 2, e.g., spinal cord velocity range 7.6 ± 3 mm/s vs. 5.6 ± 1.4 mm/s, *p* = 0.001; CSF velocity range 56 ± 21 mm/s, vs. 42 ± 10 mm/s, *p* < 0.001). These findings were further supported by adjusted data analysis. CSF stroke volume remained consistent (2.2 ± 0.8 ml vs. 2.2 ± 0.7, *p* = 0.992, adjusted data: 3.8 ± 0.8 vs. 3.7 ± 0.7, *p* = 0.063).

**Table 2:** CSF and spinal cord velocity data and adjusted data in arbitrary units (a.u.). Significant differences between

		Healthy (*n* = 70)	Patients with confirmed leak (*n* = 117)	Patients with confirmed leak and Bern score ≤ 4 (*n* = 48^+^)	Ventral leak (54%)	Lateral leak (20%)	CSF–venous fistula (20%)	Sacral leak (6%)	No leak confirmed (*n* = 8)
Spinal cord	Velocity range (mm/s)	5.6 ± 1.4	7.6 ± 3**	6.5 ± 2	7.2 ± 2.9**	8.4 ± 3.3**	8.2 ± 3.1**	6.6 ± 2.0	7.4 ± 3.1
	Adjusted (a.u.)	7.9 ± 1.4	9.9 ± 3.0**	8.9 ± 2.5	9.5 ± 2.9**	10.5 ± 3.3**	10.9 ± 3.2**	8.9 ± 2.2	9.9 ± 3.3
	Total displacement (mm)	0.7 ± 0.2	1.0 ± 0.5**	0.8 ± 0.4	0.9 ± 0.5*	1.2 ± 0.5**	1.1 ± 0.5**	0.7 ± 0.3	1.0 ± 0.5
	Adjusted (a.u.)	1.3 ± 0.2	1.6 ± 0.5**	1.4 ± 0.4	1.5 ± 0.5	1.7 ± 0.5**	1.7 ± 0.5**	1.3 ± 0.3	1.6 ± 0.6
CSF	Velocity range (mm/s)	42 ± 10	56 ± 21**	53 ± 17*	56 ± 17**	63 ± 8**	54 ± 23	52 ± 18	43 ± 14
	Adjusted (a.u.)	61 ± 9	75 ± 20**	72 ± 17*	74 ± 17**	80 ± 27**	76 ± 14*	70 ± 17	64 ± 10
	Total displacement (mm)	10 ± 3	12 ± 5**	12 ± 4	12 ± 4*	14 ± 6**	13 ± 7	11 ± 5	11 ± 3
	Adjusted (a.u.)	11 ± 3	14 ± 5**	13 ± 4	13 ± 4**	15 ± 6**	14 ± 7	12 ± 5	11 ± 3
	Flow rate—range (ml/s)	9 ± 3	10 ± 3*	10 ± 3	10 ± 3	12 ± 3**	9 ± 4	9 ± 3	9 ± 2
	Adjusted (a.u.)	12 ± 3	13 ± 3*	13 ± 3*	13 ± 3	15 ± 3**	13 ± 3	13 ± 3	13 ± 2
	Stroke volume (ml)	2.2 ± 0.7	2.2 ± 0.8	2.2 ± 0.7	2.1 ± 0.7	2.7 ± 1.0*	1.9 ± 1.0	1.9 ± 0.7	2.2 ± 0.5
	Adjusted (a.u.)	3.7 ± 0.7	3.8 ± 0.8	3.9 ± 0.7	3.9 ± 0.7	4.3 ± 0.9**	3.7 ± 1.0	3.5 ± 0.8	3.7 ± 0.5

SIH patients and controls are indicated as *p < 0.05 and **p < 0.01

+ 60% ventral leak, 20% lateral leak, 10% CSF–venous fistula, 10% sacral leak

SIH patients with a Bern score of 4 points or less (all types of spinal CSF leaks) exhibited a tendency toward increased dynamics with significantly enlarged CSF velocity ranges (53 ± 17 mm/s vs. 42 ± 10 mm/s, *p* < 0.05).

**Fig. 3:**
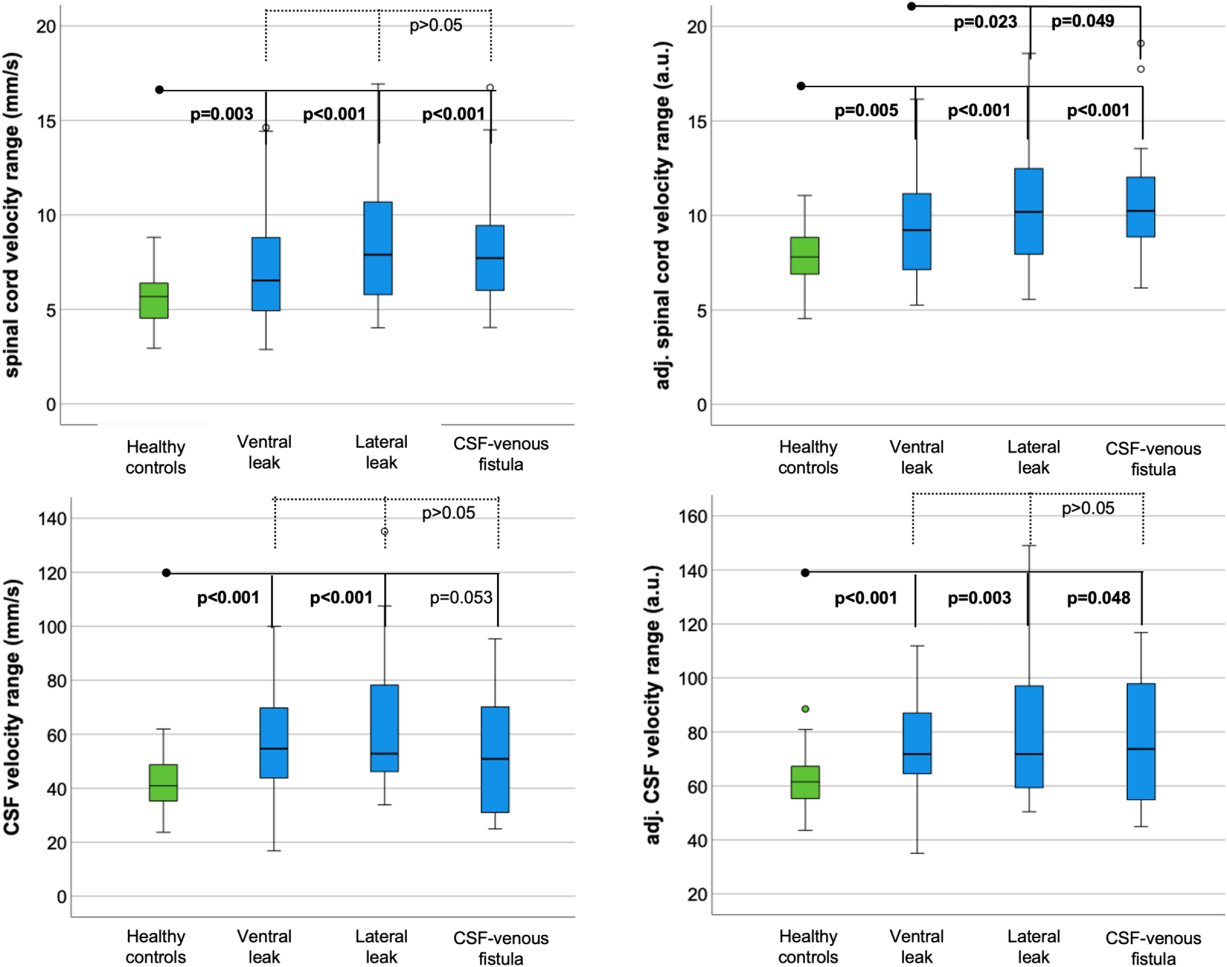
Boxplots of velocity ranges (top) and adjusted velocity ranges (bottom) in healthy participants (green) and SIH patients (blue) stratified by leak type

Subgroup analysis stratified by the type of spinal CSF leak revealed the most pronounced increase in spinal cord motion among patients with lateral leaks and CSF–venous fistulas, e.g., spinal cord velocity range 8.4 ± 3.3 mm/s and 8.2 ± 3.1 mm/s, respectively, vs. 5.6 ± 1.4 mm/s in healthy controls, *p* < 0.001, respectively (Table 2, Fig. 2). When adjusted for age and sex, spinal cord velocity range was even larger in lateral leaks and CSF–venous fistulas than in ventral leaks (*p* = 0.023, *p* = 0.049, respectively; Fig. 3).

All CSF velocity data was highest in patients with lateral leaks.

Higher Bern scores correlated with larger adjusted spinal cord velocity range and total displacement with moderate goodness of fit (correlation coefficient (95%CI) 0.44 (0.23–0.66, adjusted *R*^2^ = 0.16, correlation coefficient (95%CI) 0.068 (0.03–0.10), adjusted *R*^2^ = 0.13, *p* < 0.002, respectively, Supplement 2). Correlation analysis with CSF data showed only weak goodness of fit (adjusted *R*^2^ < 0.06, Supplement 2). Higher segmental narrowing at C2/C3 (aSCOR) as potential surrogate of brain sagging correlated with the extent of spinal cord velocities and displacement with a poor to moderate goodness of fit (correlation coefficient (95%CI) 0.25 (0.15–0.34), adjusted *R*^2^ = 0.20, correlation coefficient (95%CI) 0.03 (0.01–0.05), adjusted *R*^2^ = 0.01, *p* < 0.002, respectively). The duration of symptoms did not correlate to any dynamic data.

**Fig. 4:**
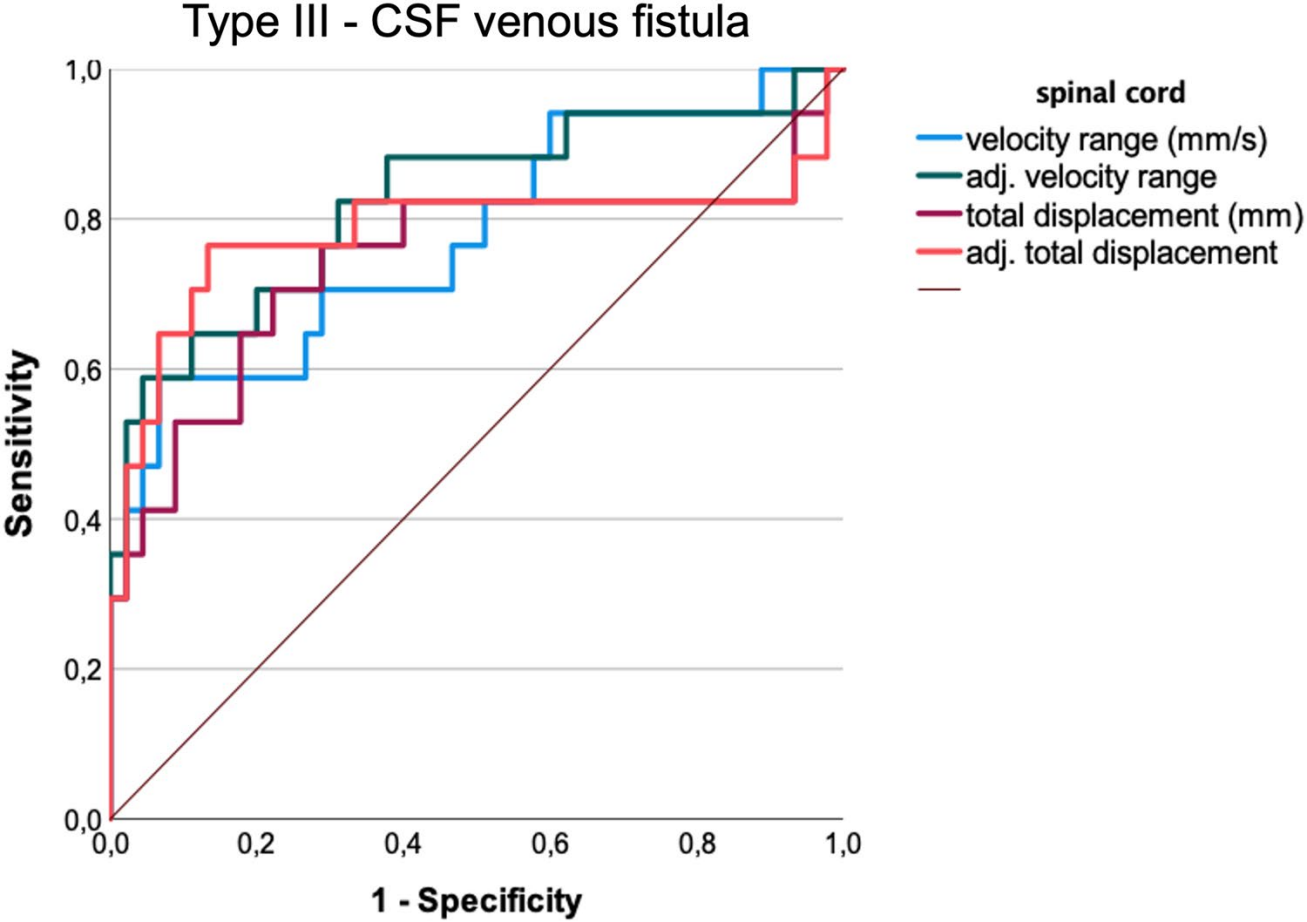
Receiver operating characteristics of spinal cord motion analysis at the segment C2/C3 in SIH patients with confirmed CSF–venous fistulas. The area under the curve (AUROC) for all spinal cord data is > 0.744, maximum is reached by measurements of the velocity range adjusted to age and sex: 0.83 in patients with CSF–venous fistula

For all 117 patients with confirmed CSF leak, encompassing all different leak types, acceptable differentiation between these patients and healthy cohorts was achieved using CSF velocity range and by adjusted CSF velocity range (AUROC 0.740, 0.747, respectively, Table 3). Discrimination by spinal cord velocity range was nearly acceptable (AUROC 0.699). Thresholds were chosen at the lowest value achieving a specificity of ≥ 90% (Table 3). Given these thresholds, discrimination was favorable across all spinal cord measurements in patients with CSF–venous fistulas (> 0.74) (Fig. 4) with sensitivity ranging between 53 and 77%, and specificity between 85 and 93%. If spinal cord velocity range was adjusted for age and sex, an excellent discrimination was achieved (AUROC 0.826, sensitivity 58%, specificity 91%).

**Table 3:** Receiver operating characteristics (AUROC) analysis/sensitivity (sens.) and specificity (spec.) non-adjusted thresholds should be adapted to age by about 10% per each decade exceeding the age of 45 years [25]

	Patients with definite leak (*n* = 117)			CSF–venous fistula (*n* = 23)		Definitive leak and Bern score < 5 (*n* = 48*)
	Threshold	AUROC	sens./spec	AUROC	Sens./spec	AUROC
Spinal cord—velocity range (mm/s) (male/female)	> 7.5 (7.8/7.0)	0.699 (0.690/0.713)	45 / 92% (49/91%/46/92%)	0.775 (0.743/0.801)	58/93% (38/89%*/58/93%)*	< 0.6
Adjusted (a.u.)	> 9.8	0.690	44/91%	0.826	58/91%	< 0.6
spinal cord—total displacement (mm) (male/female)	> 0.92 (1.0/0.91)	0.665 (0.602/0.683)	45/92% (43/90%/45/92%	0.744 (0.697/0.772)	53/91% (62/89%/42/81%)	< 0.6
Adjusted (a.u.)	> 1.5	0.641	48/91%	0.782	77/85%	< 0.6
CSF—velocity range (mm/s) (male/female)	> 56 (56/55)	0.740 (0.782/0.724)	43/92% (53/93%/43/92%)	< 0.6		< 0.6
Adjusted (a.u.)	> 71	0.747	52 / 91%	0.646	53 / 89%	< 0.6
CSF—total displacement (mm) (male/female)	> 14.0 (13.9/12.9)	0.652 (0.720/0.654)	30 / 92% (43/93%/35/92%)	< 0.6		
Adjusted (a.u.)	> 14.4	0.660	38 / 91%	< 0.6		
Flow rate—range (ml/s)		< 0.6		< 0.6		
Adjusted (a.u.)		< 0.6		< 0.6		
Stroke volume (ml)		< 0.6		< 0.6		
Adjusted (a.u.)	> 4,4	0.613	25/9%	< 0.6

### Report of non-resolved cases

Table 4 contains the history and data of the remaining cases admitted due to suspected SIH (7 cases), or formerly confirmed SIH (1 case). Dynamic data was rated as positive/negative ( ±) according to the thresholds (Supplement 2).

**Table 4:** History of patients with suspected leak due to prior signs in the MRI, or based on patient’s history; dynamic data are rated as positive if > thresholds indicating a specificity of ≥ 90%, VR—velocity range, TD—total displacement

History and signs before admission	History and signs during workup				Clinical path
	Symptoms	MRI		Myelography	
New, severe orthostatic headache, dizziness, hearing impairment starting 10 months ago, improvement after non-targeted EPB, recurrence of symptoms 3 months after EBP, SLEC +	Improvement of symptoms, but persistent orthostatic headache in the morning and second half of the day	Bern score	0	Not performed	Suspected rebound hypertension, Improvement after treatment with acetazolamide, follow-up
		SLEC	Negative		
		Spinal cord (VR/TD)	Negative/ negative (reduced values)		
		CSF (VR)	Negative		
Spontaneous new orthostatic headache for 6 months, dizziness, hearing impairment, some improvement after non-targeted EBP	Persistent symptoms preventing work	Bern score	1	Bilateral Myelography negative	Repeated non-targeted EBP, further improvement reported
		SLEC	Negative		
		Spinal cord (VR/TD)	Positive/ positive		
		CSF (VR)	Negative		
Spontaneous new orthostatic headache starting 3 months ago, Bern score 4	Spontaneous recovery	Bern score	3	Not performed	Has not reported to the clinic since
		SLEC	Negative		
		Spinal cord (VR/TD)	Negative/negative		
		CSF (VR)	negative		
Orthostatic headaches, bilateral hygroma	Inconsistent orthostatic headaches with dizziness, and tinnitus	Bern score	4	Bilateral Myelography negative	Suspected CVF, non-targeted EBP and follow-up pending
		SLEC	Negative		
		Spinal cord (VR/TD)	Negative/positive		
		CSF (VR)	Negative		
Orthostatic symptoms for 3 years: headache, dizziness, cognitive impairment, pronounced root cysts in former MRI	Persistent symptoms	Bern score	5	Bilateral Myelography: possible small tubular elongation T9/10 left	Suspected CVF, targeted EBP root cyst without improvement, repeated non-targeted EBP with incomplete resolution, follow-up
		SLEC	Negative		
		Spinal cord (VR/TD)	Positive/positive		
		CSF (VR)	Positive		
Spontaneous orthostatic headache developed 8 months ago, initial MRI Bern score 8, possible SLEC (poor quality of MRI), slow improvement after EBP	Further improvement	Bern score	4	Bilateral Myelography negative	EBP offered, patient declined
		SLEC	Negative		
		Spinal cord (VR/TD)	Negative/positive		
		CSF (VR)	Negative		
Orthostatic headache for 2 months, bilateral chronic subdural hematoma	Orthostatic headache	Bern score	5	Bilateral Myelography negative	Improvement after twist-drill trephination with drain
		SLEC	Negative		
		Spinal cord (VR/TD)	Negative/negative		
		CSF (VR)	Negative		

## Discussion

In this study, we report findings from a substantial cohort of patients with confirmed spontaneous spinal CSF leaks, presenting with increased spinal cord and CSF velocity data at the upper cervical spine when compared to healthy controls assessed by non-invasive MRI.

The data affirms the formerly published results of a proof-of-concept study [24] across all known leak types associated with SIH [20–22]. The pulsatile back and forth movements of the cerebrospinal fluid and spinal cord are part of the physiological volume shifts between the intracranial and spinal compartments (Monro–Kellie doctrine) [28]. In the case of CSF loss, a reduced resistance and therefore increased velocities, and in the case of increased cerebrospinal fluid volume, a reversed, dampened dynamic were assumed. In this context, is it crucial to note that within the compartments not only CSF volume (and pressure) adapt, but also the blood volume, especially by extension of the venous bed. Therefore, measurements of dynamic parameters within the spinal compartment are most likely a reflection of some, but never all alterations.

Within the currently presented new data, most importantly, a notable increase in spinal cord dynamics has been revealed among patients with lateral leaks, and with CSF–venous fistulas possibly serving as an additional, objective biomarker especially for the latter. This is a promising sign toward this upcoming cohort with CSF–venous fistulas that is less likely depicted, and yet least understood. The findings within this group is of even further importance, as a recent study has found CSF–venous fistulas in 56% of the entire cohort of 57 patients who presented with Bern score between zero to two, considered to pose a low risk of spinal CSF loss. [29]

Based on the current dataset, the dynamic differences between leak types cannot be explained. The anatomy of the underlying leak (lateral disruption of the transverse circular fibers of the dura mater vs. rupture of the longitudinal and more elastic fibers [30, 31]), as well as differences in leak compensation by the extension of the epidural plexus might play a role. Increased elastance of the craniospinal system in a cohort mainly consisting of patients with CSF–venous fistulas has been described [32]. This implies a reduced compliance of the craniospinal system, which might therefore account for the comparably less increased CSF velocities in this cohort.

The observed age disparity on patients diagnosed with CSF–venous fistulas, being older compared to those with other leak types, aligns with impressions gathered among specialized centers, and corroborates a recent study reporting a similar mean age of 59 years. [33] Given the previously described age-related changes in CSF and spinal cord velocities, the use of adapted data assumes significance. [25]

The moderate relation between spinal cord motion and the Bern score, which encompasses but is not limited to signs of brain sagging, suggests a potential, yet moderate link. It might be hypothesized that increased craniocaudal spinal cord motion per heartbeat may reflect a component of the overall induced mechanic stress to the adherent meningeal tissues caused by spinal CSF loss that are discussed to be involved within the pain circuits in SIH [14]. Nevertheless, it is crucial to note that these results were obtained in supine positioning within the MRI scanner, and the response of the spinal cord dynamics in an upright position remains unknown. The observed altered spinal cord motion is not unique to SIH: increased motion of the brain stem and cerebellar tonsils has been observed in Chiari type I [34], and focally increased spinal cord motion is described at the level of the cervical spinal stenosis [35, 36]. The localized narrowing of the subarachnoid space and the transduced effects of the pulsatile expansion of the vessels within this narrowed space are postulated as potential origin of increased craniocaudal motion [36–38]. In SIH, a relative decrease in subarachnoid CSF space at the upper cervical spine likely due to brain sagging has been described. [24]

Current data indicates evidence of larger spinal cord motion across all leak types, with an overlap between patients and healthy controls, particularly evident among patients with ventral leaks. However, this observation should not contradict the general hypothesis. Normal or even low values among SIH patients might be due to the individual compensatory mechanisms such as increased CSF production rates and/or augmented venous volume in the epidural venous plexus, considering that many SIH patients have normal to even increased intracranial pressure [8]. Therefore, this introduced method does not serve as an exclusionary tool for spinal CSF leaks, but offers additional information, particularly valuable in patients with CSF–venous fistulas and potentially in unresolved cases.

Regarding diagnostic pathways, non-invasive dynamic measurements could supplement the current screening process, reducing diagnostic blind spots. Exploring dynamic alterations trough non-invasive investigations in SIH and related disorders could aid in understanding the underlying dynamic mechanisms, monitoring the disease course, and identifying possible (rebound) hypertension, as suggested by preliminary data. [39]

As discussed before, assessments of spinal cord motion are more feasible compared to CSF regarding technical aspects, which further underscores its potential use (about 10% of the spinal cord measurements, and about 20% of all CSF measurements were excluded). The currently chosen method of data curation favored a restricted approach at this stage of exploration by excluding those with atypical curves even in the absence of clear artifacts and segmentation problems. At the individual level, most cases were excluded due to common phase-contrast MRI artifacts, of which some could be overcome by applying individual Venc settings (aliasing).

As a limit, there is a general bias within most SIH cohorts pertaining to this study: atypically affected SIH patients without predominant orthostatic headache and/or patients with low Bern scores are yet less likely to be admitted to centers for further investigation. Thus, patients positive for SLEC and patients with high Bern scores are the predominant groups in most SIH cohorts. Whether increased spinal cord motion could enhance the sensitivity and specificity of the Bern score remains to be explored, given the current limited number of cases with low or intermediate Bern scores negative for SLEC.

In addition, many patients were pretreated with therapies that interfered with the epidural space. As these treatments took place outside the center, the vast majority of patients had no data available, e.g., on the amount of blood applied to a blood patch. It cannot be ruled out that these pre-therapies also had an impact on the dynamic effects.

## Conclusion

This study reinforces the observation that spinal cord and CSF velocities are elevated in patients with SIH, encompassing those with CSF–venous fistulas. Essentially, patients with CSF–venous fistula exhibited notably larger spinal cord dynamics compared to healthy controls. These findings suggest that non-invasive phase-contrast MRI measurements could potentially evolve as a biomarker offering additional diagnostic value, guiding the diagnosis and treatment in SIH patients without evidence of spinal epidural fluid collection.

### Supplementary Information

Below is the link to the electronic supplementary material.

Supplementary file1 (DOCX 379 KB)

Supplementary file2 (DOCX 18 KB)

## Data Availability

Raw data can be obtained from the main author upon reasonable request.

## Abbreviations

AUROC: Area under the curve of receiver operating characteristic
BMI: Body mass index
CSA: Cross-sectional area
SD: Standard deviation
SIH: Spontaneous intracranial hypotension
SLEC: Spinal longitudinal extradural (fluid) collection
