# Supplementary material for: Non-invasive biomarkers for spontaneous intracranial hypotension (SIH) through phase-contrast MRI
Source: J Neurol. 2024 Apr 21;271(7):4336–47. doi: 10.1007/s00415-024-12365-6 (PMC11233306; doi:10.1007/s00415-024-12365-6)
Supplement: Supplementary file 1 — Supplementary file1 (DOCX 379 KB) [file 415_2024_12365_MOESM1_ESM.docx]

**Supplement 1**

**Details MRI sequences:**

T2-weighted (T2w) 3D sequence in sagittal orientation, cervical spine:

spatial resolution 0.6 mm x 0.6 mm x 1.0 mm, TR 1500 ms, TE 134 ms, Flip angle 105°, GRAPPA factor: 3, acquisition time 3:53 min

axial, 2D phase-contrast sequence CSF:

prospective ECG-triggering, spatial resolution 0.9mm x 0.9mm x 5mm, FoV 200x200 mm^2^, TR = 20.2 ms, TE = 7.7 ms, flip angle 15°, bandwidth 488 Hz/Pixel, PEAK-GRAPPA acceleration^1^, velocity encoding parameter 10cm/s, velocity vector in cranio-caudal direction, acquisition of 40 timepoints per cardiac cycle, slight alterations depending on the heart rate; approximated acquisition ~1.5 minutes

axial, 2D phase-contrast sequence spinal cord:

prospective ECG-triggering, spatial resolution 0.9mm x 0.9mm x 5mm, FoV 200x200 mm^2^, TR = 20.2 ms, TE = 7.7 ms, flip angle 15°, bandwidth 488 Hz/Pixel, PEAK-GRAPPA acceleration^1^, velocity encoding parameter 5 cm/s, velocity vector in cranio-caudal direction, acquisition of 40 timepoints per cardiac cycle, slight alterations depending on the heart rate; approximated acquisition time ~1.5 minutes

^1^Jung B, Ullmann P, Honal M, Bauer S, Hennig J, Markl M: Parallel MRI with extended and averaged GRAPPA kernels (PEAK-GRAPPA): optimized spatiotemporal dynamic imaging. J Magn Reson Imaging 2008, 28(5):1226.

**Details MRI data processing**

Segmentation and data analysis were fully automated based on separately trained 3D hierarchical deep convolutional neural networks (CNN) applying the in-house platform NORA (www.nora-imaging.org, (1)).

The training procedures and implementations have been described before (2) (3), in short: CSF-space and spinal cord tissue had been manually segmented by two independent, fully trained physicians in 15 to 20 randomly chosen datasets of the anatomical images and the phase-contrast magnitude images (SB, MH, KW). These segmentations served as ground truth to train separate CNNs that each were trained to segment CSF-space and spinal cord tissue of the 3D T2-weighted, and the magnitude images of the phase-contrast sequences, respectively. The quality of the training was assessed by Dice coefficients >0.9 (4) (5). The CNN generated a voxel-wise likelihood indicating the probability of each voxel to belong to the structure in question (given in 0 to 1; CSF, and spinal cord tissue, respectively). An additional CNN was trained analogously to detect and annotate cervical vertebrae.

Anatomical data: Regions of interest (ROI) were defined to include the central third of the intervertebral space (C2/C3 to C7/T1), which covers the narrowest parts of the CSF space along the cervical spine where degeneration is typically most pronounced. All detected voxels with a likelihood of 0.8 and higher were included in the analysis. The mean cross-sectional area (CSA) of all slices included on the ROI was given in mm^2^ per intervertebral segment.

The test-test reliability of data assessments rated by intra-class correlation coefficient (ICC, single measures, two-way mixed effects model, absolute agreement) has been reported at an excellent level (ICC >0.98) per parameter and segment (3).

Dynamic data: Axial ROIs were designed to include all voxels detected at a likelihood of 0.9 to belong to spinal cord tissue, and of 0.7 to belong to CSF, respectively. An automated correction of possible aliasing effects was implied within the software (7) (8). Phase-drift correction was performed by subtraction of the median velocity per sequence.

| Axial phase-contrast MRI C2/C3 | Test-test ICC  (peak-tp-peak amplitude / total displacement) | Scan-rescan ICC (peak-tp-peak amplitude / total displacement) |
| --- | --- | --- |
| Spinal cord | 0.966 / 0.924 | 0.967 / 0.966 |
| CSF | 0.992 / 0.995 | 0.957 / 0.951 |

ICCs per sequence and segment

**Data exclusion based on shape of the curves:**

The individuals’ velocity curves were visualized over one heartbeat irrespective of the duration by automated interpolation of the data to 30 timepoints in NORA. In case of obvious outliers (indicated by stars) compared to otherwise typical patterns, the data was excluded (Figure)


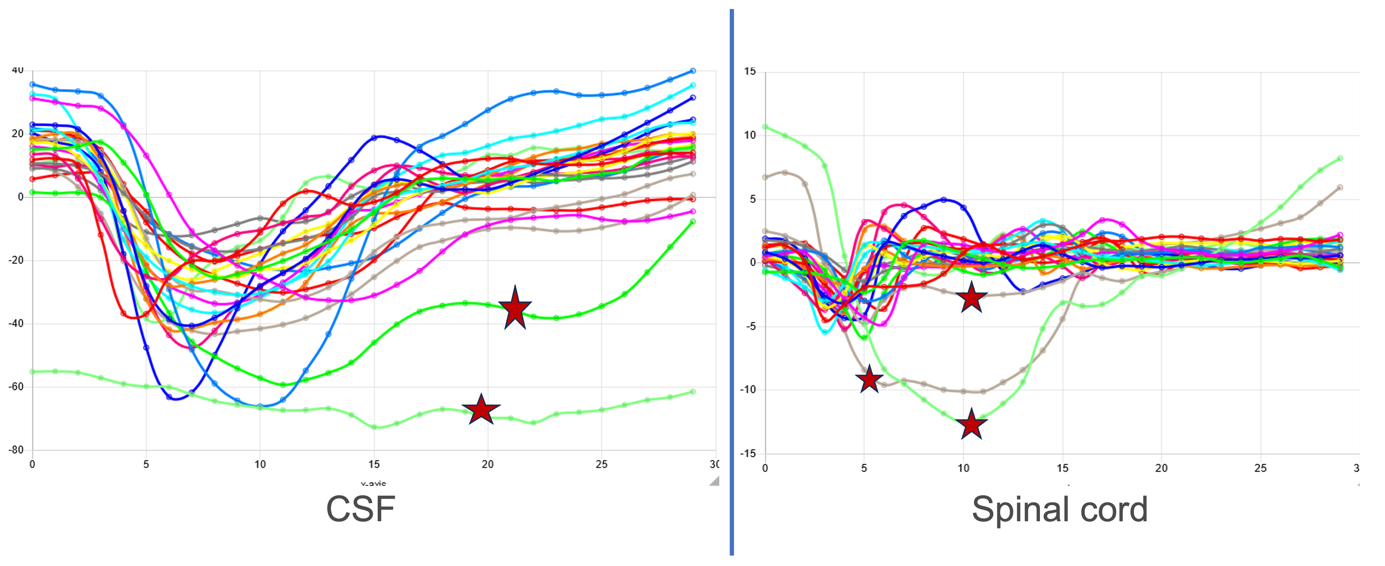


References:

1. nora-imaging. [Online] http://www.nora-imaging.org.

2. Wolf K, et al.. CSF flow and spinal cord motion in patients with spontaneous intracranial hypotension (SIH): A phase-contrast MRI study. *Neurology. 2022 Nov 10:10.1212/WNL.0000000000201527. doi: 10.1212/WNL.0000000000201527. Epub ahead of print.*

3. Wolf K, et al. Focal cervical spinal stenosis causes mechanical strain on the entire cervical spinal cord tissue – a prospective controlled, matched-pair analysis applying phase-contrast MRI. *NeuroImage:Clinical epub ahead of print, doi: 10.1015/j.Nicl.2021.102580.* 2021.

4. De Leener B, Cohen-Adad J, Kadoury S. Automatic Segmentation of the Spinal Cord and Spinal Canal Coupled With Vertebral Labeling. IEEE Trans Med Imaging. 2015 Aug und 34(8):1705–18.

5. Chen M, et al. Automatic magnetic resonance spinal cord segmentation with topology constraints for variable fields of view. Neuroimage. 2013 Dec und 83:1051–62.

6. Wymer DT, et al. Phase-Contrast MRI: Physics, Techniques, and Clinical Applications. Radiographics. 2020 Jan-Feb und 10.1148/rg.2020190039., 40(1):122-140. doi:.

7. Johnson KM, Markl M. Improved SNR in phase contrast velocimetry with five-point balanced flow encoding. *Magn Reson Med.* 2010, Bd. 63, S. 349-355.

8. Wolf K, et al. Spinal Cord Motion in Degenerative Cervical Myelopathy: The Level of the Stenotic Segment and Gender Cause Altered Pathodynamics. *J Clin Med.* 10.3390/jcm10173788 (2021)
