# Supplementary material for: Non-invasive biomarkers for spontaneous intracranial hypotension (SIH) through phase-contrast MRI
Source: J Neurol. 2024 Apr 21;271(7):4336–47. doi: 10.1007/s00415-024-12365-6 (PMC11233306; doi:10.1007/s00415-024-12365-6)
Supplement: Supplementary file 2 — Supplementary file2 (DOCX 18 KB) [file 415_2024_12365_MOESM2_ESM.docx]

Supplement 2:

|  | healthy | | SIH patients | | p |
| --- | --- | --- | --- | --- | --- |
|  | Mean | SD | Mean | SD |  |
| aSCOR C2/C3 | 23.93 | 4.6o | 28.27 | 5.65 | <0.001 |
| aSCOR C3/C4 | 29.88 | 4.96 | 32.76 | 5.33 | 0.011 |
| aSCOR C4/C5 | 31.14 | 5.06 | 32.64 | 5.28 | 0.447 |
| aSCOR C5/C6 | 31.74 | 4.43 | 33.19 | 7.18 | 0.325 |
| aSCOR C6/C7 | 29.82 | 4.38 | 30.32 | 6.14 | 0.946 |
| aSCOR C7/T1 | 24.49 | 5.41 | 24.21 | 5.11 | 0.815 |

Anatomical data of healthy participants and SIH patients.

|  | Adjusted data (arbitrary units) | Spinal cord | | CSF | | | |
| --- | --- | --- | --- | --- | --- | --- | --- |
|  |  | velocity range | Total displacement | velocity range | Total displacement | Range CSF flow rate | Stroke volume |
| Bern Score | R | 0.41 | 0.39 | 0.27 | 0.23 | n.s. | n.s. |
|  | Adj. R^2^ | 0.15 | 0.13 | 0.05 | 0.04 |  |  |
|  | p | <0.001 | 0.001 | 0.029 | 0.034 |  |  |
|  | B (95%CI) | 0.44 (*0.23-0.66)* | 0.068 (*0.03 - 0.10)* | 2.07 (*0.51 – 3.91)* | 0.43 (*0.08 – 0.77)* |  |  |
|  | p | 0.001 | 0.004 | 0.016 | 0.025 |  |  |
| aSCOR | R | 0.49 | 0.32 | 0.43 | 0.34 | 0.30 | n.s. |
|  | Adj. R^2^ | 0.20 | 0.01 | 0.18 | 0.11 | 0.08 |  |
|  | p | <0.001 | 0.002 | <0.001 | 0.001 | 0.004 |  |
|  | B (95%CI) | 0.25 (0.15-0.34) | 0.03 (0.01 - 0.05) | 1,72 (0.99 – 2.56) | 0.36 (0.15 – 0.56) | -0.18 (-0.31- -0.06) |  |
|  | p | <0.001 | 0.001 | <0.001 | 0.001 | 0.004 |  |

*Data of the multiple linear regression model on the spinal cord and CSF dynamic measurements adjusted to age, sex, and to CSF space narrowing if applicable. Bern score. R – correlation coefficient, B – regression coefficient, CI – confidence interval, n.s. – not significant*
